# Supplementary material for: The COVID-19 Pandemic and Responses in Nursing Homes: A Cross-Sectional Study in Four European Countries
Source: Int J Environ Res Public Health. 2022 Nov 19;19(22):15290. doi: 10.3390/ijerph192215290 (PMC9696230; doi:10.3390/ijerph192215290)
Supplement: Supplementary file 1 [file ijerph-19-15290-s001.zip › ijerph-1980350-supplementary.pdf]

Supplementary Table S1. Definitions of the prevention and control measures taken by the nursing homes. CluDe Study, 2020.

Preventive and control measures	Definitions
**Visitor ban**	Banning visitors from accessing the nursing home premises.
**Residents’ containment**	Confining the residents to their room.
**Residents’ sectorization**	Partitioning areas/spaces of the nursing home premises with clear restrictions on who can access it.
**Dedicated COVID-19 unit**	A COVID-19 unit is an area where COVID-19 positive tested patients are taking care of. COVID-19 unit was implemented when there were at least one confirmed case of COVID-19 in the nursing home.
**Residents’ screening**	Systematic testing of the residents.
**Staff screening**	Systematic testing of the staff members.
